# Editorial: Identification of therapeutic targets and novel biomarkers in prostate cancer volume II

**DOI:** 10.3389/fonc.2023.1163235

**Published:** 2023-03-06

**Authors:** Shashwat Sharad, Alagarsamy Srinivasan

**Affiliations:** ^1^ Department of Research, Rajiv Gandhi Cancer Institute and Research Center, Delhi, India; ^2^ NanoBio Diagnostics, West Chester, PA, United States

**Keywords:** prostate cancer, biomarker, diagnosis, prognosis, therapy, body fluids, multi-omics, tumor antigens

A glance at the history of science reveals that progress over the years has been in the form of human ingenuity combined with the development of new technologies. The field of cancer is no exception in this regard. The first study on the induction of solid tumors by a filterable agent (virus) was reported by Francis Peyton Rous in 1911 using chicken as a model system (1). The agent later was designated as Rous sarcoma virus (RSV), a member of avian retroviruses. This novel observation was completely ignored by the scientific community for several decades. Further work on RSV later by investigators led to two major findings which have been instrumental for the advances in cancer research since the mid-1970s. These findings are i) The discovery of reverse transcriptase enzyme in the retrovirus particles was a finding that challenged the central dogma of molecular biology. This enzyme was shown to carry out the synthesis of a DNA copy from viral RNA and other RNAs as templates and became an essential tool in molecular biology; ii) Further studies on RSV showed that specific viral sequences were essential for the transformation of cells in culture. The investigations on RSV ultimately led to the identification of viral oncogene and corresponding cellular oncogene and demonstrated the genetic basis of cancer for the first time (2). Thus, the oncogene revolution in cancer began with the studies on RSV, the chicken virus. Based on this information, numerous oncogenes were discovered later by using diverse retroviruses and also by direct transfection of DNA (derived from tumors/tumor cells) into cells in culture. Initial studies on ras oncogene paved the way for this line of research.

The characteristic feature of cancer cells is uncontrollable growth, unlike normal cells where the growth is under control through gene networks. Considering the unlimited replication of cancer cells, it is likely that the cancer cells may release DNA, RNA, and proteins into the body fluids possibly through the lysis of cells and/or other mechanisms. The technological advances in molecular biology, as noted above, have enabled the analysis of gene expression patterns in cancer cells in comparison to normal cells. Hence, such studies have the potential to pinpoint specific gene expression patterns in cancer cells. Similarly, specimens from cancer patients have also been the subject of DNA sequence analysis to uncover mutations such as point mutations, deletions, and rearrangements. The nature of the specimen samples from prostate cancer patients and the resulting analytes from the analytical methods are presented in **Figure 1**. The methodologies are designed to evaluate the body fluids such as blood, sera and plasma, saliva, and urine in addition to tumor tissues and tumor-derived cells. Such approaches have led to the identification and characterization of novel biomarkers for further analysis as potential tools for the diagnosis and therapy of prostate cancer. It has been suggested that the current list of blood/serum, urine, and tissue-based biomarkers requires further improvement to enhance the predictive accuracy, diagnosis, prognosis, and treatment response of prostate cancer.

**Figure 1:**
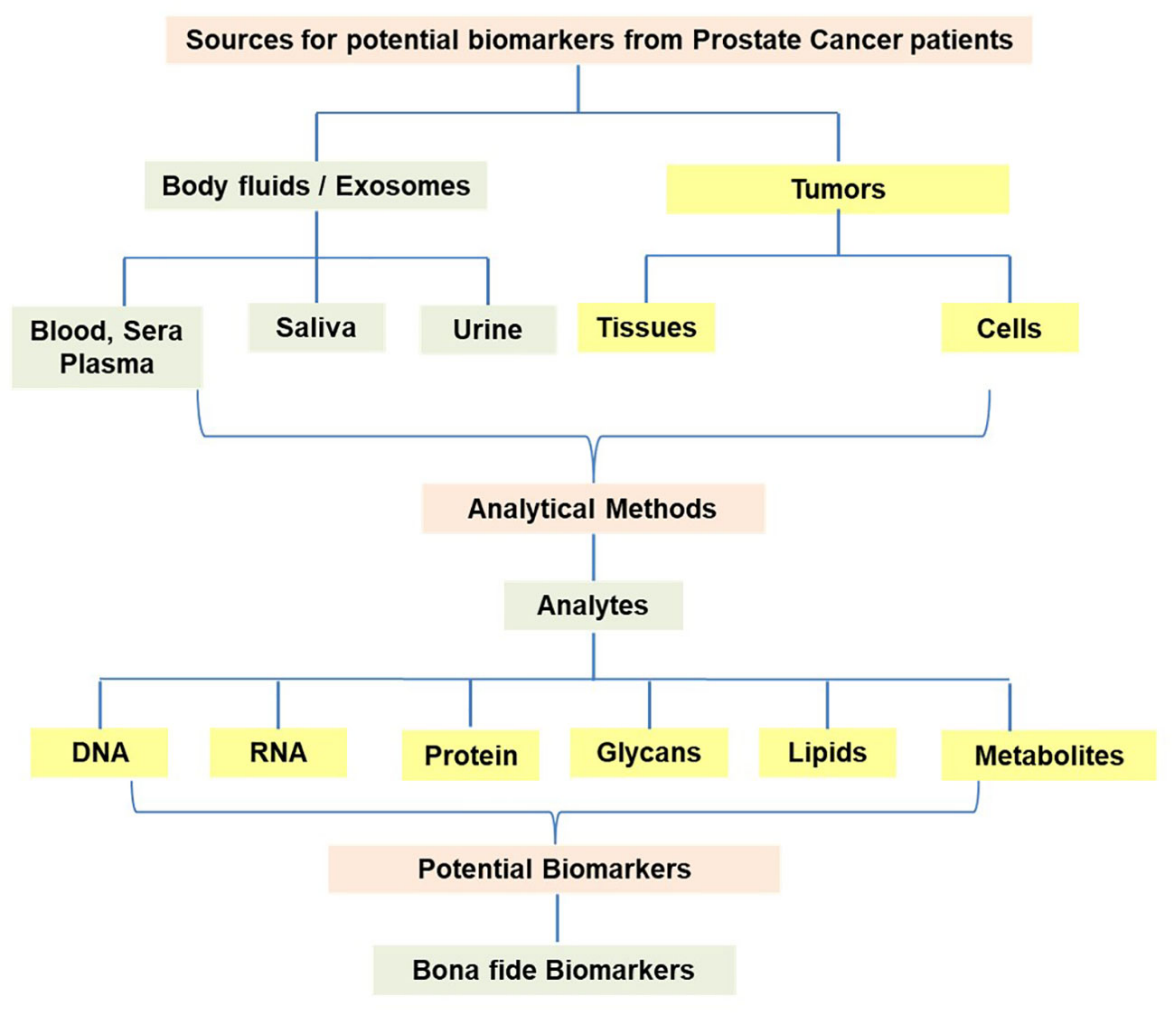
Development of novel potential molecular biomarkers from various specimens derived from prostate cancer patients.

The predictive cancer biomarkers are classified into three groups comprising screening, diagnostic, and prognostic (3). Like other cancers, biomarkers are needed for addressing the important questions in prostate cancer including the following: markers for accurate diagnosis of prostate cancer, markers with the ability to differentiate indolent from aggressive cancer, and markers that can differentiate Benign prostatic hyperplasia (BPH) from prostate cancer. There are several tests currently available in the market for prostate cancer. However, there are also limitations with some of the tests including sensitivity, specificity, the time needed for the completion of tests, and invasive approaches utilized for securing the samples for analysis. To avoid overtreatment, improve risk assessment, and provide more selective treatment, it is important to discover more precise prostate cancer biomarkers. In addition to early detection, therapeutic response, and staging of cancer, there is an urgent need for biomarkers for sub-classes of prostate cancer for effective treatment options (4).

This scenario calls for the exploration of biomarkers utilizing samples from body fluids and tumor tissues of patients. The information regarding the analytes such as DNA, RNA, proteins/peptides, Glycans, lipid profiles, and metabolites is generated through the utilization of methodologies using multiple platforms. The search for biomarkers may also include the analytes representing the responses from the host to tumor antigens released from the cancer tissues/cells. It has been shown that tumor antigens (either overexpressed or containing alterations in protein due to point mutations) have the potential to elicit antibody responses (known as autoantibodies) in the host. Hence, autoantibodies can also be explored in the plasma/sera for diagnostic and prognostic purposes.

The multifocal nature and tumor heterogeneity (intratumor and intertumoral) of prostate cancer is the biggest challenge in developing clinically treasured prostate cancer biomarkers. The genomic, transcriptomic, proteomic, and metabolomic studies not only facilitate the characterization and subtyping of prostate cancer but also enhance the understanding of the tumor microenvironment. The new biomarkers should take into account of already established clinical and pathological information variables to enhance individualized risk assessment and clinical management of prostate cancer.

## Author contributions

SS and AS wrote the editorial, made direct and intellectual contributions, and approved it for publication. The editorial is focused on the need for biomarkers that may include the analytes such as DNA, RNA, proteins/peptides, glycans, lipid profiles and metabolites in addition to autoantibodies against tumor antigens. Both the body fluids and tumor tissues/cells offer as ideal sources for prostate cancer biomarker development.

## References

[1] Rous P. A sarcoma of the fowl transmissible by an agent separable from the tumor cells. J Exp Med (1911) 13:397–411. doi: 10.1084/jem.13.4.397 19867421 PMC2124874

[2] Moore PS Chang Y. Why do viruses cause cancer? Highlights of the first century of human tumour virology. Nat Rev Cancer. (2010) 10(12):878–89. doi: 10.1038/nrc2961 PMC3718018 21102637

[3] Kohaar I Petrovics G Srivastava S. A rich array of prostate cancer molecular biomarkers: Opportunities and challenges. Int J Mol Sci (2019) 20(8):1813. doi: 10.3390/ijms20081813 31013716 PMC6515282

[4] Adamaki M Zoumpourlis V. Prostate cancer biomarkers: From diagnosis to prognosis and precision-guided therapeutics. Pharmacol Ther (2021) 228:107932. doi: 10.1016/j.pharmthera.2021.107932 34174272

